# Whole-Genome Sequence of Geobacillus thermoleovorans ARTRW1, Isolated from Armutlu Geothermal Spring, Turkey

**DOI:** 10.1128/MRA.00269-20

**Published:** 2020-06-11

**Authors:** Merve Oztug, Anil Cebeci, Hande Mumcu, Muslum Akgoz, Nevin Gul Karaguler

**Affiliations:** a Istanbul Technical University, Faculty of Science and Letters, Department of Molecular Biology and Genetics, Istanbul, Turkey; b National Metrology Institute, TUBITAK UME, Kocaeli, Turkey; c Halic University, Faculty of Science and Letters, Department of Molecular Biology and Genetics, Istanbul, Turkey; University of Delaware

## Abstract

The thermophilic microorganism Geobacillus thermoleovorans ARTRW1 was isolated from water samples collected in the Armutlu hot spring in Turkey. Here, the whole-genome sequence and its annotations are reported.

## ANNOUNCEMENT

Geobacillus thermoleovorans is a thermophilic Gram-positive bacterium isolated from various environments, including air (1), hot springs (2, 3), a gold mine (4), and sugar refineries (5). The Geobacillus thermoleovorans strain ARTRW1 is a rod-shaped, Gram-positive, and aerobic bacterium isolated from water samples collected from the vent of the Armutlu hot spring (74.9°C, pH 7.1). Briefly, the water samples were collected in 50-ml sterile tubes and transferred to the laboratory within 3 h. The GPS coordinates of the sample collection site are 40.545557°N, 28.841777°E. Next, 1 ml of water sample was used to inoculate 50 ml of Luria-Bertani (LB) liquid medium and was incubated at 60°C for 72 h. The culture was streaked onto LB nutrient broth solidified with Gelzan (0.4% by mass) and incubated at 60°C for 24 h. A single colony was picked and purified by repeated streaking (five times) onto fresh culture plates. The strain was grown on the LB liquid medium at 60°C overnight prior to the DNA isolation. The genomic DNA was isolated using the Promega Wizard genomic DNA purification kit according to the manufacturer’s protocol. A TruSeq Nano DNA low-throughput library prep kit (Illumina, USA) was used to construct the sequencing libraries. Quality control for the size distribution and quantity of the libraries created was performed using a 2100 bioanalyzer (Agilent Technologies, USA). Synthesis sequencing (SBS) was performed using a HiSeq rapid SBS kit v2 (Illumina) with single fragment readings (2 × 150-bp paired-end [PE] reads) obtained by splicing the two fragments end to end. The cBot v2 system (Illumina) was used to construct clusters by bridge amplification, and the Illumina HiSeq 2500 platform was used for sequencing. A total of 17,383,190 sequences were generated.

The Geneious Prime v2019.1 program was used to control the quality of the sequences. The paired ends were merged using the BBmerge tool, duplicate reads were removed using the Dedupe tool, and the BBNorm tool was used for error correction and normalization. The Geneious assembler method was used to map the reads to the Geobacillus thermoleovorans CCB US3 UF5 reference genome obtained from the NCBI, and a consensus sequence was formed. Reads whose quality was lower than 30 were filtered out during the mapping process using the Geneious assembler method. Default parameters were used for all tools. The consensus assembly generated a single sequence of a circular genome with 3,596,341 bp (50-fold coverage). The average chromosome G+C content was 52.27%. From the annotation of Geobacillus thermoleovorans strain ARTRW1 using the software PGAP with GeneMarkS, 3,885 coding sequences (CDS), 4,002 genes, and 88 tRNAs were found (6). In addition, 1,381 unidentified hypothetical protein sequences (hypothetical protein CDS) were found in the 5,835 CDS. The average nucleotide identity (ANI) value was calculated using EzBioCloud TruBAC ID software v1 (7), revealing 98.32% similarity to the Geobacillus thermoleovorans group. The ANI value with respect to the Geobacillus thermoleovorans CCB US3 UF5 strain was calculated to be 99.90% using EzBioCloud ANI calculator v1 (8). The Comprehensive Antibiotic Resistance Database (9) was searched for the presence of antibiotic resistance genes, and no match for resistance genes was found.

### Data availability.

The genome sequence of Geobacillus thermoleovorans ARTRW1 has been deposited at DDBJ/EMBL/GenBank under the accession number CP042251. The associated BioProject and BioSample accession numbers are PRJNA556596 and SAMN12360852, respectively. The raw reads were deposited in the Sequence Read Archive under the SRA accession number SRX7882959.
